# A fixed reconstruction of fully edentulous patients with immediate function using an apically tapered implant design: a retrospective clinical study

**DOI:** 10.1186/s40729-020-00271-1

**Published:** 2020-11-23

**Authors:** Mehmet Akif Eskan, Gokhan Uzel, Seyit Yilmaz

**Affiliations:** 1 Clinic Eska, Polat Tower, Fulya Mah., Yesilcimen Sk. Sisli, Istanbul, Turkey; 2 College of Dental Medicine, Department of Periodontics, NOVA Southeastern University, Fort Lauderdale, FL USA; 3 Eve-Dent, Sakarya, Turkey

**Keywords:** Dental implants, Total edentulism, Primary implant stability, Tapered, Immediate function

## Abstract

**Background:**

Immediate function has become an accepted treatment modality for fixed restorations in completely edentulous jaws. It is known that implant microtopography (surface) may enhance osseointegration, while implant macrotopography (macrodesign) plays an important role in primary stability in the patient requiring an immediate loading. The aim of this retrospective study was to evaluate the clinical and radiographic outcomes of the edentulous subjects treated with narrow and/or regular diameter, which placed and loaded immediately.

**Methods:**

Forty-two consecutive patients received 171 implants, including regular and narrow diameter implants (NDIs). Each jaw, 19 mandibles and 24 maxillae, was treated with a fixed-full arch prosthesis according to the Straumann® Pro Arch concept. The majority (95%) of the restorations were supported by four implants, of which the posterior two implants were tilted. A provisional functional acrylic prosthesis was delivered on the day of surgery. All patients were followed up to 55 months. Cumulative survival rate was determined using Kaplan-Meier analysis. Radiological measurement of marginal bone level was performed.

**Results:**

The overall follow-up time for survival rate was up to 55 months. Four implants (3 implants in maxilla, 1 implant in mandible) were lost, resulting in an overall cumulative implant survival rate of 97.7%. Implant survival rate in the axial and tilted implants was not statistically significant. The mean of interproximal marginal bone loss was 0.15 mm after 24 months. Good soft tissue health was observed in almost 99% of patients. The final prosthesis survival rate was 100%.

**Conclusions:**

The results of this retrospective pilot study indicated that total edentulous patients requiring an immediate implant placement and loading can be successfully treated with this implant design. The improved mechanical properties of these implants might give a more conservative treatment option for the jaws showing a severe horizontal alveolar bone resorption.

**Supplementary Information:**

The online version contains supplementary material available at 10.1186/s40729-020-00271-1.

## Background

Current implant research is focusing on developing safe and cost-effective surgical and prosthetic protocols for the treatment of completely edentulous patients [1]. Immediate function is one of several treatment concepts that have been receiving much attention recently. Besides the development of improved treatment protocols, investigation is continuing to focus on new implant designs to increase predictability in clinically demanding situations [2]. It is well known and documented that the implants micro and macrodesign play an important role for the osseointegration [3, 4]. The results of numerous studies unanimously have suggested that microtextured implant surfaces create favorable conditions to enhance osseointegration of dental implants compared to smooth surface implants [3]. Recently, researchers are focusing on implant macrodesign, which is one of the factors that influence implant stability [5]. Initial implant stability, a key factor influencing the implant survival rate [2, 6], might be difficult to be achieved in soft bone or fresh extraction sites. This primary stability allows the implant to mechanically adapt to the alveolar bone until osseointegration is achieved [7]. Lack of primary implant stability compromises the osseointegration process [8]. The success of primary stability depends on bone quantity, bone quality, surgical technique, and/or implant macrodesign [6]. Most implants originally had a parallel-walled design, but these were not necessarily appropriate in many cases, especially in soft bone. One of the biggest disadvantages of the parallel-walled cylindrical implants was that they increased the risk of labial bone perforations [9]. Therefore, tapered implants were introduced not only to reduce labial plate perforations but also they can help to enhance primary implant stability [8].

Tapered implants have been shown to exert a certain amount of lateral compressive force on the surrounding cortical bone [10]. Additionally, narrow implant tips with aggressive threads extending to the apex may facilitate osteotomy and thus aid the surgeon during implant insertion in underprepared sites. This feature can result in increased the implant primary stability, thereby extending the indications for immediate loading. Bone level tapered (BLT) implants are designed with an apically tapered implant body and self-tapping threads to support under-preparation of the alveolar bone. Therefore, they can help to achieve a high primary stability in soft bone or fresh extraction sockets where primary stability is needed to at least 30 Ncm [11].

Vertical and/or horizontal alveolar bone resorption mostly follows tooth loss and results in an alveolar ridge deficiency [12], thus making implant placement difficult. When the alveolar bone width is insufficient to insert a regular size implant (diameter < 4 mm) in the posterior area, additional surgical techniques may be required for bone regeneration [13]. To avoid these extra surgical steps, it could be plausible to use narrow diameter implants (NDIs < 3.5 mm). However, there is a concern regarding the fatigue strength of these types of implants, especially in the posterior area, which is exposed to high biting forces [14, 15]. To overcome these problems, titanium alloys with higher tensile and yield strength have been made by different companies, such as a new titanium-zirconium alloy (Ti-Zr) comprising 13–15% zirconium [16]. This enhanced biomechanical Ti-Zr alloy has good biocompatibility and allows for the use of NDIs in clinically challenging conditions since their survival rate has been shown to be very similar to others [15, 17].

Therefore, the aim of this study was to retrospectively evaluate the performance of implants sizing 3.3 or 4.1 mm of diameter in the fixed treatment of total edentulous patients, requiring an immediate implant placement and loading.

## Methods

**Fig. 1:**
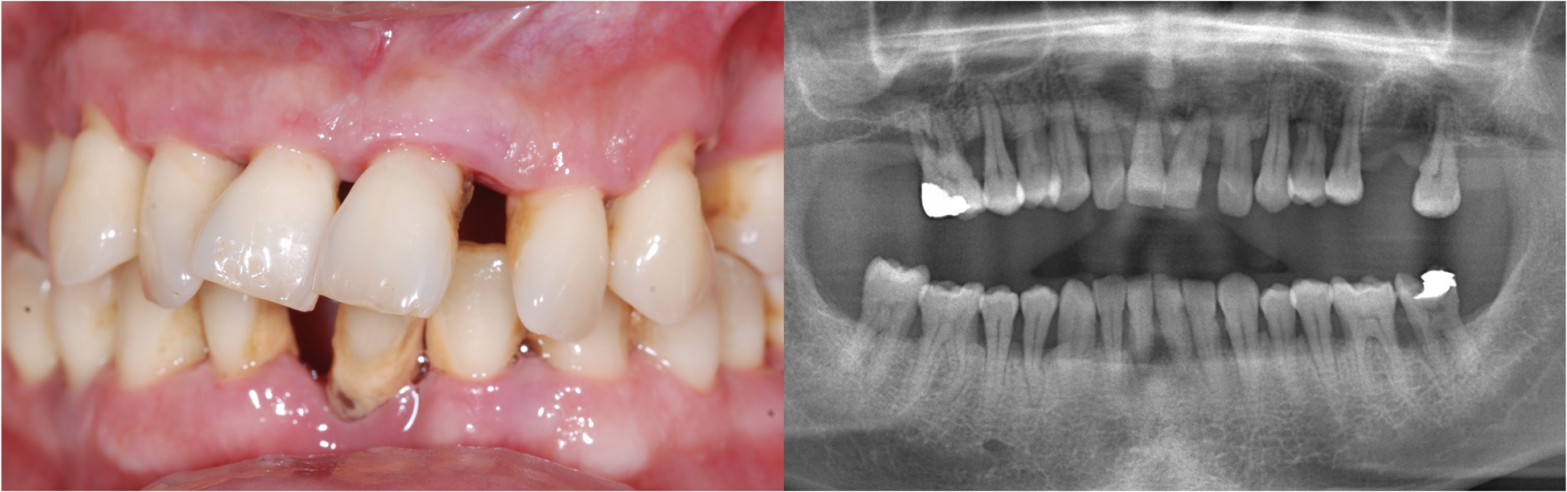
Clinical images of 42-year-old male patient receiving a full-arch maxillary reconstruction. Preoperative intraoral picture (right) and orthopantomography (left) showed the need for full-arch prosthetic reconstruction of the maxilla

This multicenter study was conducted in two different private clinics in Turkey, Clinic Eska (Istanbul) and Ev-Dent (Sakarya). Forty-two patients (from April 2015 to August 2016) were included for this retrospective analysis. This study was approved by the University of Uskudar institutional review board in 2018. It was also conducted in accordance with the Helsinki Declaration of 1964, as revised in 2013. The subjects included in this study were complete edentulous or had teeth with hopeless prognosis [18]. Prognosis for the non-extracted teeth was good to fair [18]. Periodontal treatments, including surgical and non-surgical, were done on these teeth. The patients with a hopeless prognosis and bad oral hygiene went through a gross oral debridement one week before the surgery to avoid a possible contamination during the surgery. Subject’s teeth with hopeless prognosis (Fig. 1) were extracted at the day of the surgery, and immediate implant placement was done on these patients. The BLT implant system (Straumann® BLT SLA® Roxolid® Basel, Switzerland) has regular and narrow diameter implants. A total of 171 BLT implants were placed, supporting 43 fixed full-arch prostheses (24-maxilla, 19-mandible) (Table 1). Eleven patients showed a total (upper and lower) edentulism. Twenty-one patients presented only single jaw edentulism, either maxilla (15 jaws) or mandible (6 jaws).

**Table 1:**
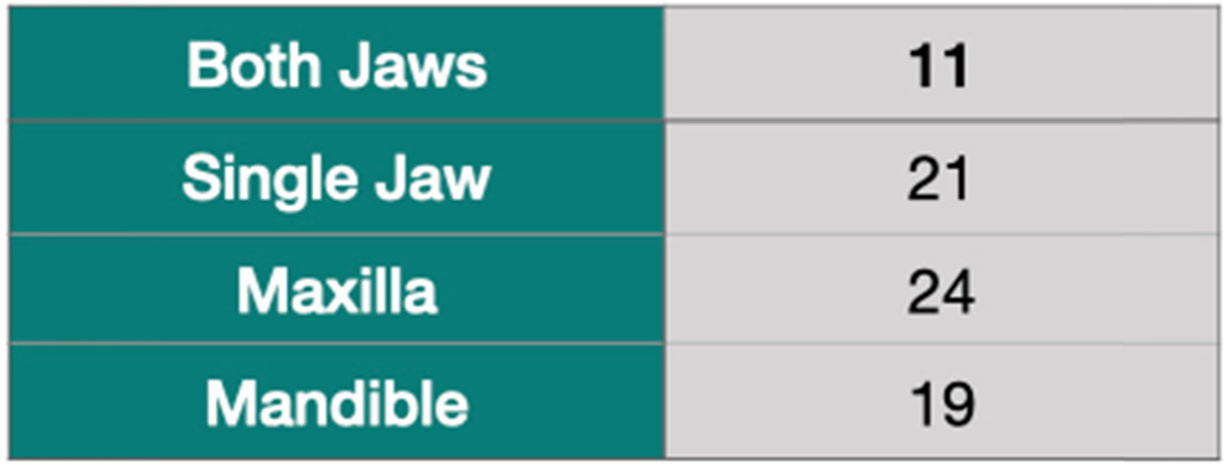
Distribution (maxilla and/or mandible) and the number of treated jaws

### Inclusion and exclusion criteria

The rationale for patient selection was to include all patients who received a full-arch reconstruction during a specific time interval at the clinic. This time interval was chosen so as to include the very first patient who received this treatment and all consecutive patients treated in the same way up to a given date, which allowed for the collection of at least 24 months of follow-up data for the orthopantomography (OPG) evaluation. The patients treated were in need of full-arch rehabilitation and presented a bone situation amenable to the placement of at least four implants. The minimum bone width and height were required at least 4 mm and 8 mm, in each patient, respectively. Exclusion criteria were active infection at the intended sites of implant placement, chemotherapy or radiotherapy within the 12 months before surgery, uncontrolled diabetes, or hematologic disease.

Radiographic screening was performed using OPG and cone beam computed tomography. A careful clinical examination of the patients was performed assessing jaw size and relations, bone volume, and occlusal relations. The patients included at the Clinic Eska and Ev Dent clinics were treated by surgeons (MAE and SY, respectively).

### Surgical procedures

The surgical procedures were performed under local anesthesia which comprised articaine hydrochloride and epinephrine (0.012 mg, Safoni-Aventis Deutschland GmbH, Germany). Antibiotics (amoxicillin 1 g) was given twice daily on the day before surgery and then daily for 7 days. Anti-inflammatory medication (ibuprofen, 400 mg) was given for 3 days postoperatively starting on the day of surgery. Following the extraction of hopeless teeth, any sharp edges were smoothed, and all sockets were debrided before implant placement. Implant placement was assisted by a specially designed surgical guide (Straumann® Pro Arch Guide) to facilitate correct implant tilting and accurate positioning of the implants in relation to each other. Screw-retained abutments (torqued to 25–30 Ncm) were placed in relation to the opposite jaw. The implants were placed according to standard procedures except that under-preparation was used when needed to get a final torque of at least 30 Ncm. Countersinking was used only when needed to create a space for the head of tilted implants. A bone profiler was then used to create a space for multiunit implants, especially for the tilted implants. The shortest axial and tilted implants measured 8 and 10 mm, respectively (Table 2). The diameter of the implants in the anterior or posterior region was determined according to the width of the alveolar crest. Upon the alveolar ridge width deficiency observed, NDIs were also used in the posterior region (Table 3). The function of the prosthesis has been reported that it was not dependent the number of implant inserted [19], and it has been shown by the others that a total of four implants in each jaw in the total edentulous patient resulted in a good survival rate for a long time [20]. The majority of the jaws received four implants. When one of the implants in each jaw did not reach the 30 Ncm primary stability, an extra implant was placed. The alveolar bone width, as mentioned above, and height determined the implant size. The shortest implant used in this study was 8 mm. However, it was not used any short implant (< 8 mm) in the posterior region and the tilted implants were always longer than the axial placed implants. The tilted posterior implants angulation was corrected with 30° multi-unit abutments (Institut Straumann AG). In the anterior area, the implants were introduced with either 0 or 17° multi-unit abutments (torqued to 30 Ncm). In 3 weeks after the maxillary surgery, his lower jaw treatments including implant placements on #23, 26 (3.3 × 10 mm, BL Straumann), 28 (4.1 × 10 mm SP Straumann).

**Table 2:**
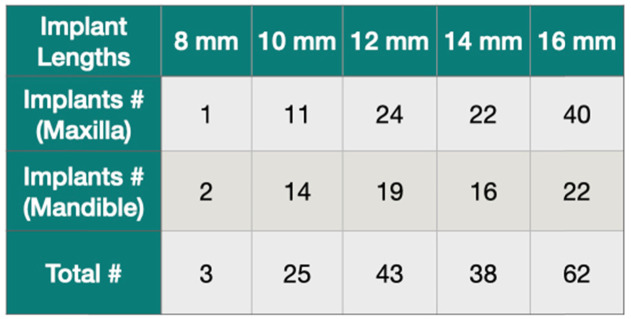
Number of implants inserted and their lengths

**Table 3:**
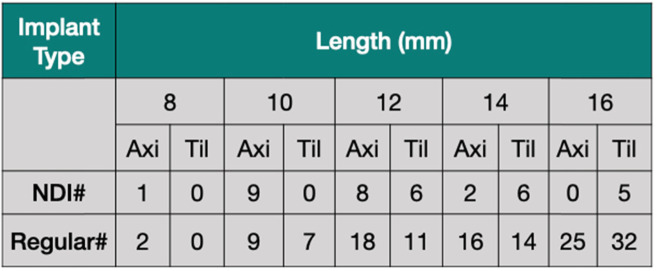
Number of NDIs (3.3 mm of diameter) and regular implants (4.1 mm of diameter) placed. Anterior implants were placed axially (Axi), and posterior implants were placed by tilting (Til) around 30°

### Delivery of provisional and final prostheses

**Fig. 2:**
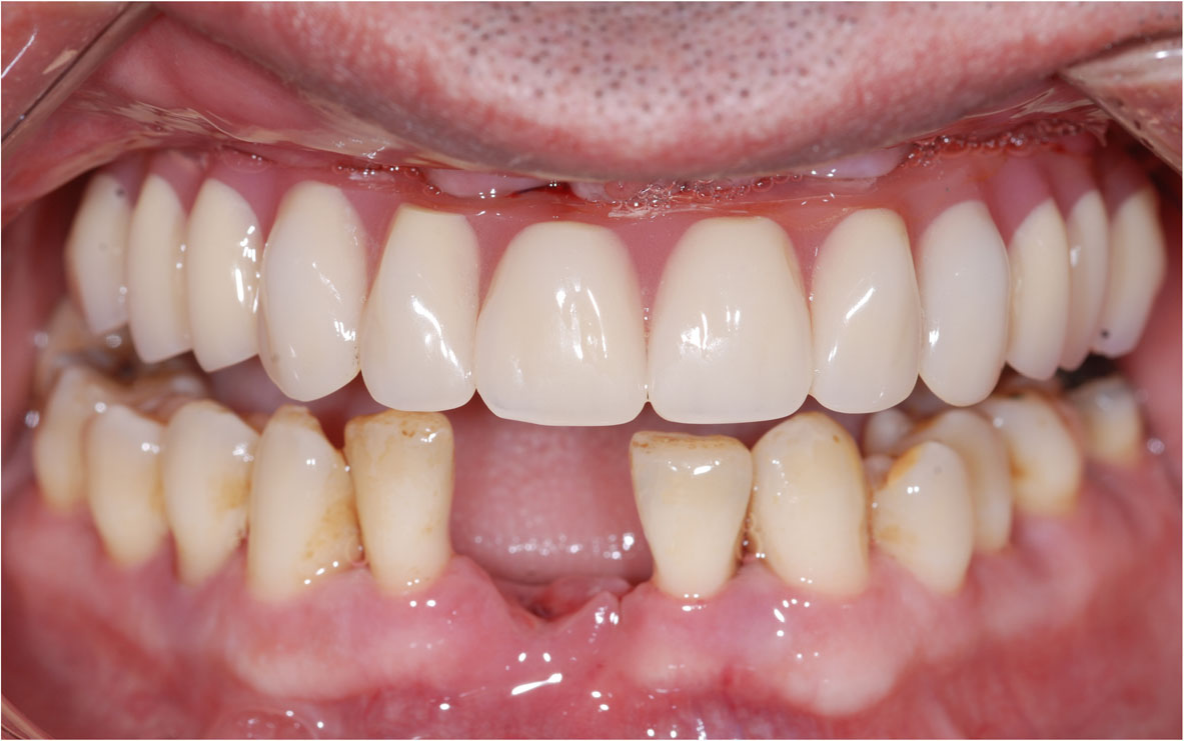
Intraoral clinical view of the provisional maxillary prosthesis after abutment connection

Healing caps (Institut Straumann AG) were placed over the screw-retained abutments. The mucosa was then sutured with 4.0 absorbable sutures (Chromic Gut, Salvin, Charlotte, NC, USA). Provisional full-arch acrylic prostheses were delivered on the day of surgery. A small volume of bite registration silicone was placed on a previously made full-arch denture. This prosthesis was then seated on the healing caps to estimate implant positions in the oral cavity. After making holes in the provisional prosthesis, the healing caps were removed and temporary titanium copings (Institut Straumann AG) were placed on the abutments. The holes in the acrylic provisional prosthesis were filled with self-curing acrylic. The patient was asked to close their mouth in centric relation. The provisional prosthesis was removed from the patient’s mouth, trimmed, and polished. No later than 3 h after surgery, an acrylic provisional with 10 teeth was delivered (Fig. 2). Occlusal screws were torqued to 10 Ncm. Regardless of the patient, a night guard made from hard acrylic plates using a standard vacuform were delivered to each patient at the day of surgery.

**Fig. 3:**
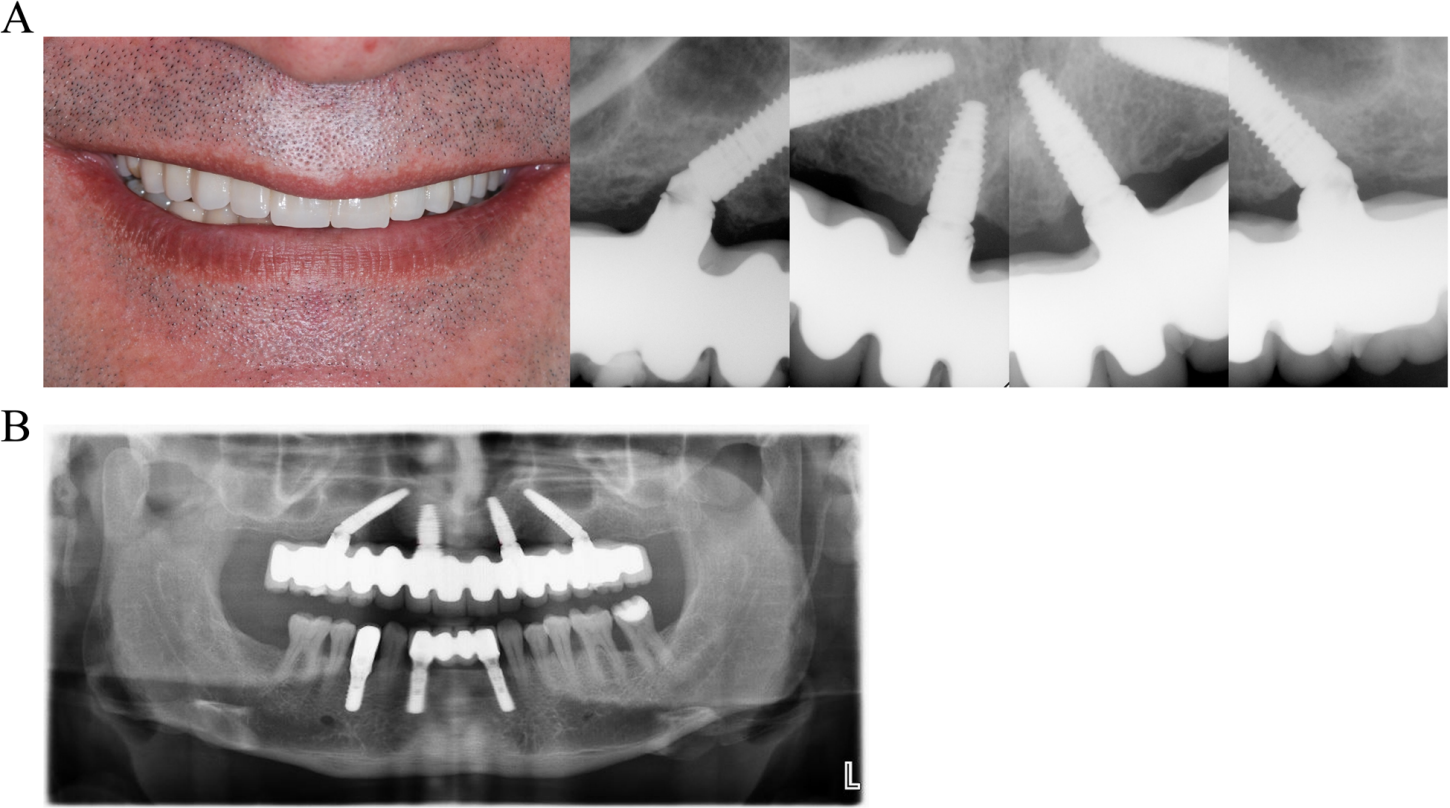
A view of the final ceramic prosthetic reconstruction (left) and periapical radiographs (right) of the case at 42 months of follow-up (**a**). The OPG view at the 42 months (**b**)

After 4 months of healing, an impression was taken from all jaws using an open tray technique, which is considered to be superior to closed-tray techniques [21]. The definitive prostheses were fabricated using CAD-CAM, or laser sintering in some cases, and made from metal and ceramic or acrylic materials (Fig. 3a). The final prosthesis usually comprised 12 teeth. The maximum cantilever on each side was no more than 10–12 mm (Fig. 3b).

In order to ensure no occlusal overloading of implants, especially the NDIs, surgical and prosthetic cautions were taken. Surgically, tilted implant (distal implants) was not tilted more than 30° by utilizing the Pro Arch Guide (Straumann) since more than 30° inclination was shown to increase occlusal overload [22]. Prosthetically, flat fossa and grooves for wide freedom in centric, a narrow occlusal table, shallow occlusal anatomy, and reduced cuspal inclination were obtained in both the provisional and final prosthesis, since they have been shown to reduce occlusal overloading [23]. Occlusal scheme, regardless of regular or NDI supported prosthesis, was obtained with no working, nonworking, or protrusive interference contacts. The canine protected occlusion was obtained in all cases. Additionally, acrylic resin was used as an occlusal material to provide a shock absorbing mechanism [24]. Periodic recalls for the subjects used NDI was given every other month in the first year. After that, they were recalled twice a year to ensure a tight recall schedule and monitor the patients closely.

### Implant survival

The primary outcome of this study was to calculate implant survival rate (up to 55 months). An implant was considered successful (100% survival rate) if fulfilled function of supporting full-arch restoration, stable when tested clinically, no radiolucency on radiographs, and no peri-implant inflammation or suppuration [5]. Therefore, the implant survival rate was determined based on Malo’s criteria [5].

### Soft tissue assessment

Peri-implantitis, peri-implant mucositis, fistula, bleeding on probing, suppuration, and numbness of the lower lip or chin were evaluated [25–27].

### Marginal bone level

Mesial and distal peri-implant marginal bone level was measured using OPG taken right after surgery and during follow-up visits, such as 0, 4, 12, and 24 months. All radiographs were taken using Romexis software (Planmeca, Helsinki, Finland). The marginal bone level (the most coronal bone-to-implant contact) was assessed on mesial and distal aspects using Romexis software. All panoramic radiographs were calibrated using an inserted implant length. Mesial and distal values were then measured from each implant on panoramic radiographs taken on day 0 and at the most recent follow-up visit, taking the implant neck as a reference for each measurement. All measurements were done by calibrated dental assistants (BD and SC). To calibrate the blinded dental assistants, they were asked to measure the inserted implant length, from at least three different patients, by using the same software (Romexis). All taken measurements were evaluated based on the actual implant length. Once they had accomplished a correct measurement, they were asked to measure all bone loss. Patients included in the bone level analysis needed to have undergone OPG at surgery and follow-up time points. In total, readable radiographs were obtained from 86% of the patients and measurements were recorded at 0, 4, 12, and 24 months.

### Prosthesis success

A prosthesis was considered a failure if function was compromised for any reason. Fracture of the implant or any prosthetic component was recorded, as were technical complications with the abutments or other prosthetic components.

### Statistical analysis

Cumulative implant survival rate was determined using the Kaplan-Meier statistical analysis, based on the unit per reconstruction. Log-rank analysis was done to compare the survival proportion between two jaws, maxilla and mandible.

## Results

### Implant survival

**Fig. 4:**
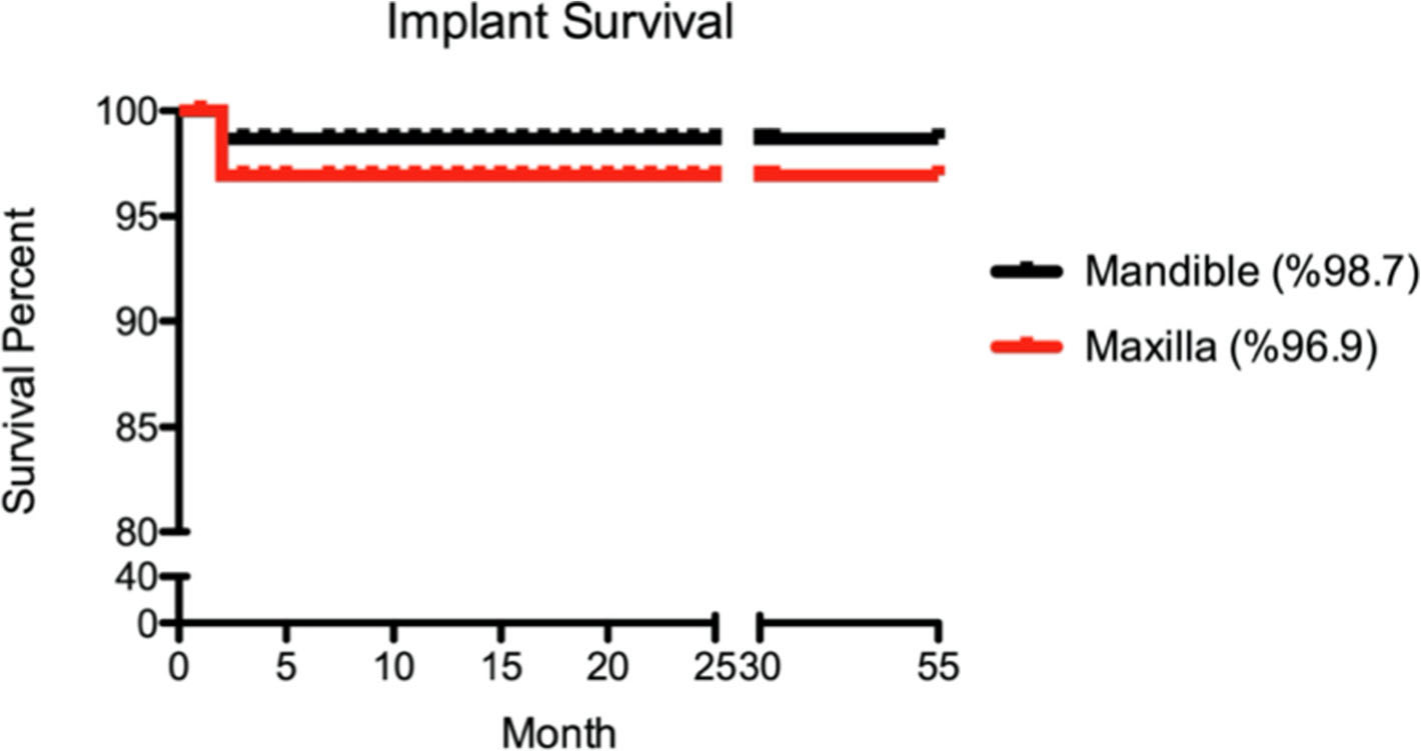
Showing survival rates in maxilla and mandible. A cumulative survival rate was 96.9% and 98.7% in maxilla and mandible up to 55 months, respectively. It was not statistically significant between the jaws based on log-rank analysis (*p =* 0.44)

All patients were followed up 32 to 55 months after the surgery. Implants used in this study had a diameter of 4.1 (134 implants) or 3.3 mm (37 implants) and measured different lengths (Table 3). The shortest and longest implants measured 8 and 16 mm, respectively. A total of 171 implants (98 in maxilla, 73 in mandible) were placed (Table 2). A total of 37 NDIs was used in this study. Seventeen of these implants were placed by tilting in the posterior area (Table 3). Eleven patients presented upper and lower total edentulism. However, 22 patients presented total edentulism in a single jaw, upper or lower (Table 1). Total of four implants failed, yielding a 97.7% cumulative implant survival rate for the study. Three implants failed in the maxilla, resulting in 96.6% survival rate. It was seen only a single implant failure in the mandible, resulting in 98.7% (Fig. 4). However, these survival rates between the jaws were not significant. All failures were seen before the final prosthesis delivery. Failed implants (4.1 × 10 and 4.1 × 16) were removed, and new implants were placed in different surgical sites or different sizes of implants.

### Interproximal marginal bone level and soft tissue

Overall, soft tissue health was good. Persistent biological problems stemming from an infected mucosa were observed at the 1-year follow-up in two patients who had not attended all their postoperative follow-up visits. Radiographic examination revealed local bone defects around two adjacent implants in one of the patients, and around one implant in the other. Clinically, implants with peri-implantitis showed swelling soft tissue and bleeding on probing. After these peri-implant problems had been detected, both patients received a rigorous hygiene maintenance treatment which prevented further bone resorption. Implant stability was maintained throughout the treatment. The implant that had a radiological bone loss but not observed any soft tissue recession was kept as it was. All OPGs, in addition to the initial OPGs, were taken in subjects at 4, 12, and 24 months (± 2 months). The mean of the bone loss from 0 to 24 months was 0.15 mm, but the mean of the bone loss between 12 to 24 months was 0.09 mm (Table 4). Furthermore, it was also reported if the mean bone loss showed any differences between the upper and lower jaw. We found that there was 0.16 mm and 0.14 mm bone loss at the maxilla and mandible in 24 months, respectively. However, the mean bone loss was detected 0 mm and 0.1 mm in the maxilla and mandible from 12 to 24 months (Table 5).

**Table 4:**
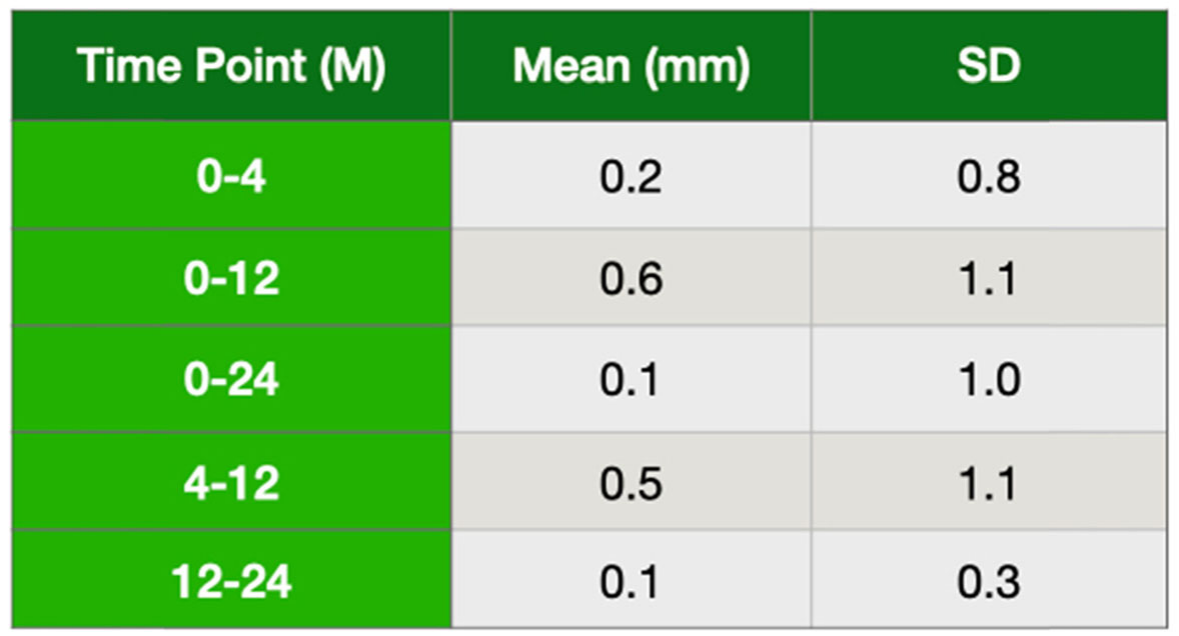
The mean of bone loss (M, month)

**Table 5:**
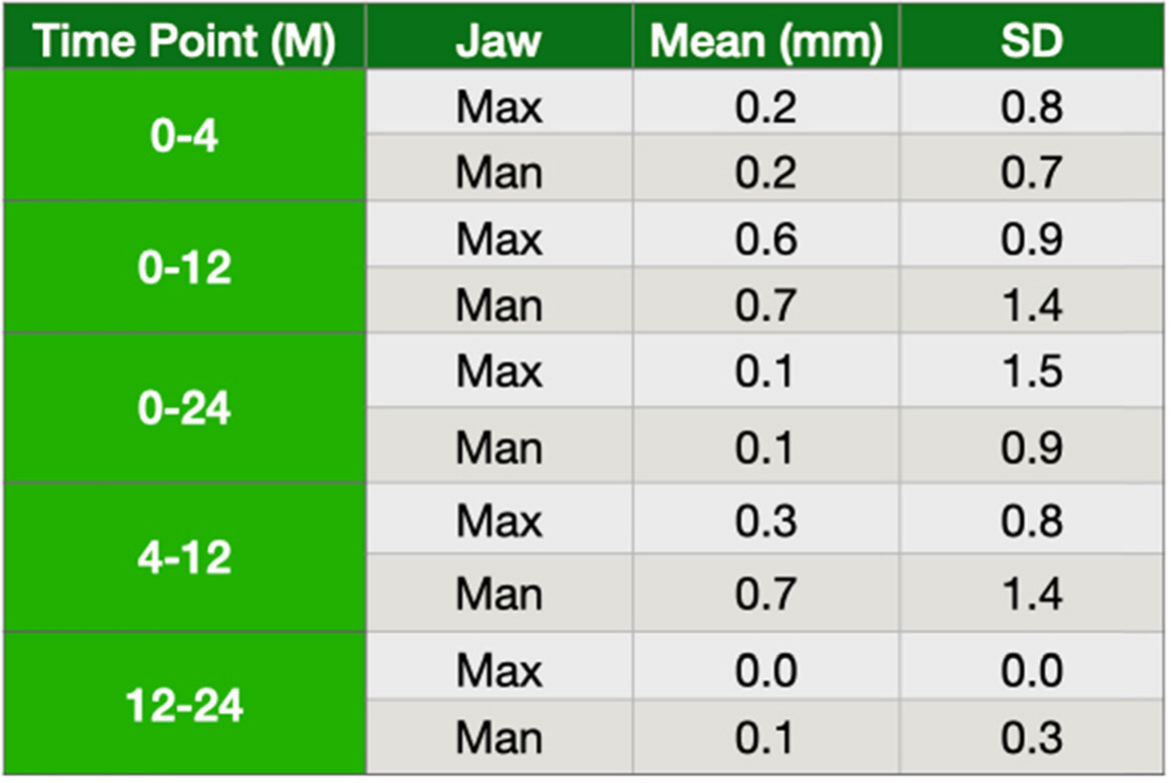
The mean of bone loss based on the jaws (M, month)

### Mechanical complications

The only mechanical complications were prosthetic screw loosening, final prosthesis fracture, and/or porcelain chipping. Screw loosening and porcelain chipping were seen in two and three patients, respectively. None of the final prostheses was broken. However, around 15% of the provisional acrylic prostheses was broken during the 4-month healing period. These broken provisional prostheses were fixed at chairside and delivered to the patient within a couple of hours. All these patients were identified as bruxers, which was probably the main cause of the screws coming loose. After retightening the screws and re-emphasizing the use of night guards, no further loosening occurred during the observation period. No implant fracture and no fracture or loosening of abutment screws was observed during the observation period.

## Discussion

This retrospective investigation showed the performance of NDIs and novel implants placed in edentulous jaws as support for immediately loaded full-arch restorations. In clinical studies, the most commonly reported parameter for evaluating the effectiveness of an implant-supported rehabilitation is the survival rate, that is, whether the implant survives in the mouth or has to be removed. A cumulative clinical survival rate (primary outcome of this study) was 97.7% up to 55 months, indicating this implant design can be used with predictable results with immediate treatment protocols in various types of bone. This survival rate was comparable to results obtained with other immediate/early loading protocols that have been reported for the same indication [20].

Four implants were lost in two patients. Our study showed implant survival rate of maxilla and mandible was 96.9% and 98.7% up to 55 months, respectively. Our results were very comparable to the others [5, 28]. Malo and colleagues found a survival rate of 98.9% at 1 year. However, our study showed a 97.7% survival rate up to 55 months (4.5 years), which is longer than their follow-up [5], suggesting that this implant design resulted in a highly comparable implant survival rate.

The implants used in this study had a narrow tip and an aggressive macrostructure facilitated insertion in underprepared sites by creating an osteotomy. This narrow implant tip rendered the BLT implants more flexible in term of vertical positioning as compare to lacing this feature. This important feature might be a valuable if it is desirable to realign the axial potion of the implant without repeating the drilling procedure.

In our study, the follow-up radiographs including periapical (Fig. 3a) and OPG (Fig. 3b) clearly demonstrated that the amount of interproximal bone resorption in tilted and axial implants was similar at the mesial and distal surfaces and consistent with other studies [28, 29]. No differences in crestal bone loss were observed between tilted and axial implants at the 4–12 and 24-month follow-up evaluations. This finding was consistent with the literature, in which tilted placed implants can function similar to axial placed implants [30, 31].

The overall interproximal bone remodeling around the inserted implants showed less than 1 mm in the first year but, but these implants showed less than 0.2 mm bone loss (Table 4) between 1 and 2 year follow-up. This finding was pretty consistent with others [5, 30]. This observation clearly indicated that this implant design might give an acceptable bone remodeling in following years. The other observation in this study was to compare if the placed implants would behave differently in the upper and lower jaw. There was 0.6 mm and 0.7 mm bone loss in the maxilla and mandible in the first year after loading, respectively. These findings suggested that interproximal bone loss was reasonable. However, the success rate could not be calculated based on the OPG readings since it gives only two-dimensional measurements, mesial and distal.

Using NDIs might lead to increased non-axial occlusal forces [24]. However, evidence has shown that nonaxial loading was not associated with an implant failure [32] and tilting of posterior implants (splinted in a full arch fixed restoration) does not result in more stress around distal implant as compared to anterior implants [33]. Occlusal overload might lead to mechanical complication, including screw loosening or fracture of the abutment or prosthesis [24]. Therefore, maintenance and periodic evaluation are especially important in order to manage potential mechanical complication on NDIs. One of the findings in this study was to observe how NDIs would behave in the full arch restorations. One of the biggest advantages of NDIs for the patient is the reduction in the duration and cost of treatment since bone grafting is required less often. It is well documented that NDIs are very useful in the partial edentulous cases that show a limited horizontal ridge width to avoid extra surgical steps, including horizontal bone augmentation [4, 6, 34, 35]. Studies regarding to the use of NDIs in the total edentulous patients are very limited. A case report showed NDI could be used to avoid extra surgical steps in full-arch rehabilitations [36]. However, this study used a 3.5-mm implant, which was larger than those used in our study (3.3 mm), and this case follow-up time was shorter (1 year) than our follow-up (up to 4.5 years). One of the issues in the usage of NDIs in the full arch rehabilitation could be an implant fracture under the biting forces for a long time period. To overcome this problem, it is plausible to use an implant with a high fatigue strength. Currently, using stronger implants made from a Ti-Zr alloy with excellent biocompatibility [37] appears to be a reliable treatment option for restoring cases with reduced crestal width (i.e., a narrow ridge) [15]. Because NDIs traditionally manufactured from grade IV Ti might result in an implant fracture, it was deemed appropriate to use stronger implants like the Ti-Zr Roxolid® implants [38]. Throughout this study, the failures were seen on the regular diameter (4.1 mm) implants but none of NDIs failed. Importantly, it was not seen any implant fracture on NDIs in 55 months follow-up, indicating that NDI implants could be safe to use in the total edentulism. However, there is a need to see how NDI would behave in the long time as compared to regular implant size diameter (> 4 mm).

Bruxism is one of the critical issues in patients requiring the full arch implant supported restorations. It is well documented that 20–35% of patients can generate biting forces causing microfracture of bone around implants resulting in implant failure [39]. It is very likely that total edentulous patients may have higher occlusal forces than patients with natural teeth due to diminished proprioception sensors. As a result of tooth extraction, the patient will not have any the periodontal ligament, which provides the central nerve system with feedback for sensory and motor [40]. However, implants only have feedback from distant mechanoreceptors resulting in almost 8-fold less tactile sensitivity than nature teeth [41]. Therefore, it may be a key point to introduce a night guard for all patients seeking a fixed implant-supported full arch restoration. Otherwise, lack of a night guard use could face not only mechanical complications, including screw loosening and/or prosthetic fractures, but it can also cause an implant failure. Patients who faced implant failure in this study presented screw loosening in their provisional prosthesis and reported that they did not use their night guards. Therefore, reminding and re-emphasizing the use of night guards to the patients may avoid more post-op complications stated above.

Regarding prosthetic complications, other authors have reported the most common complications as being prosthetic tooth fracture, tooth wear, maxillary hard relines, and screw loosening [42]. In this study too, the most common prosthetic complications were fracture of the provisional restoration, screw loosening, and porcelain chipping. In the final restorations, some prosthetic relining was also necessary. However, these kinds of problems were easily dealt with at chairside or within a few days and did not result in any major complications at implant level.

Hygiene complications were also explored in this study, because an early diagnosis of a problem in maintaining dental implant soft tissue health is necessary to reduce the prevalence of peri-implant diseases [27, 43]. Most of the hygiene problems observed in the patients were seen during the provisional phase. These patients were re-instructed in dental hygiene (by recommending the use of a water-pik), and their prostheses were checked for any concavity opposite the oral tissues. One of the key factors for avoiding plaque accumulation is that the tissue surface of the prosthesis should be well polished flat or convex.

The outcome was favorable in terms of quality of life [44] when compared with the traditional 3- to 6-month healing phase, which entails protecting the implants from premature loading [45] and further surgery to expose the implants and connect the trans-mucosal components, all of which in turn increases the time and cost of treatment as well as patient morbidity.

## Conclusion

The results of this retrospective pilot study indicated that total edentulous patients requiring an immediate implant placement and loading can be successfully treated with narrow or regular diameter implants. The improved mechanical properties of these implants might give an alternative and more conservative treatment option for the patients showing a severe horizontal alveolar bone resorption. However, surgical and prosthetic cautions should be considered for the use of NDIs in the molar area.

## Supplementary Information

**Additional file 1.**

## Data Availability

The data was provided as a supporting file.
